# Application of microtomography and petrography techniques for the characterization of porosity of synthetic carbonatic rock minerals before and after acidification processes

**DOI:** 10.1038/s41598-022-19577-8

**Published:** 2022-10-11

**Authors:** Katia Galindo, Cecília Lins, Leonardo Guimarães, Analice Lima, Katarina Silva, Abraão Nova

**Affiliations:** 1 Federal University of Pernambuco, UFPE, Recife, Pernambuco Brazil; 2 Rural Federal University of Pernambuco, Cabo de Santo Agostinho, UFRPE, Recife, Pernambuco Brazil

**Keywords:** Environmental sciences, Engineering, Materials science

## Abstract

The objective of this work is to physically characterize and analyze synthetic carbonate rocks through microtomography and petrography techniques, focusing on a comparative analysis before and after degradation with a reactive fluid. For this study, physical characterization analysis with computerized microtomography and petrography on the samples before and after the acidification procedure was performed. The petrographic analysis verified an increase in both intergranular and intragranular porosities after dissolution. The microtomography analysis quantified the maximum increase in porosity, from 11.8 to 41.3% in the two-dimensional analysis and 31.6–52% in the three-dimensional analysis of the porous structures. Furthermore, the pores were quantified according to their area, and data was obtained on the orientation of the pores, providing insight into the preferred paths of fluid flow. It was also observed that the microtomography technique was an effective tool for characterizing fractures in the samples before and after dissolution. Such analyses are crucial for the extraction and injection of fluids at high depths due to the mechanical and physical risks arising from the dissolution of minerals as well as changes in pressure, temperature, and saturation, all of which affect the stress state of the reservoir rock.

## Introduction

Energy resources such as water, gas, and oil fill the empty spaces contained in rocks. The knowledge of pore-related features is essential in the investigation of reservoirs. Rock porosity is an important topic of study due to several genetic implications. Since the experimental techniques of porosity analysis allow for a quantitative approximation but do not produce a visualization of the porous framework, petrophysical analysis has motivated the search for new analytical techniques for the study of pores. Petrographic analysis by optical microscopy allows the visualization and quantification of intergranular pores; however, it is restricted to the two-dimensional (2D) space, with the quantification being less representative. Quantitative data related to porosity and pore size distribution in rocks are determined directly in the petrographic lamina and indirectly using gas or mercury injections in pycnometers^1–3^.

Another technique for the detailed visualization of rock microstructures is microtomography. X-ray computed microtomography (μCT) was developed based on the traditional tomography procedure, but with a focus on small sample analysis. It is a non-destructive analysis method that consists of obtaining several micrographic slices and the internal three-dimensional (3D) construction of the samples, thus making it possible to determine the area and volume. This technique has been developed to facilitate analysis inside samples of undisturbed soils and rocks, mainly by evaluating the distribution of pores and minerals. Furthermore, it demonstrates the spatial configuration and nature of samples and how they can influence soil and rock behaviors as well as fluid transport processes.

Through microtomography, 3D qualitative and quantitative data related to the shape, size, distribution, volume, area, and spatial distribution of minerals, as well as pores and fractures on a microscale, can be obtained^4–9^. The spatial porosity and mineral distribution of geological materials significantly influence fluid transport processes. These processes are crucial in projects focused on the extraction and injection of fluids at high depths, due to the increase in mechanical and physical risks arising from mineral dissolution and changes in pressure, temperature, and saturation^10^. These phenomena can damage the rock and, consequently, prevent sustainable exploitation of natural resources^11–15^.

The study of phenomena involving reservoir rocks and their characterization is complex. To investigate rock–fluid interactions, X-ray μCT can be used to gather information on the microstructure of rocks. Several studies have used the X-ray microtomography method for digital image processing, such as the work by^13^, which developed a study of natural and artificially cemented limestone rocks subjected to chemical weathering. Through the petrographic images and microtomography, the internal structures of the samples were analyzed regarding the dissolution of grains caused by contact with the reactive fluid. Using this technique, the authors demonstrated pore connectivity and its quantification before and after weathering of the rock. These results made it possible to identify and quantify both in the natural and artificially cemented rocks the relationship between the micro and macro scales as well as changes in the mechanical behaviors of the samples.

^16^performed experimental studies to estimate changes in stresses and strains of synthetic carbonate rock using an edometric cell with horizontal stress measurements. The authors sought to understand hydromechanical and chemical phenomena that occurred and altered the initial characteristics of the rock. Through X-ray μCT analysis, it was possible to identify fractures inside the samples caused by the application of vertical loads together with the dissolution process, as well as the increase in porosity resulting from the dissolution of the grains. These analyses were crucial to understanding the processes that occurred when rocks make contact with reactive fluids. Acidification of reservoirs by geological storage of CO_2_ and advanced oil and gas recovery chemically alter reservoir rock.

Another application of the μCT technique can be seen in the work of^17^, which analyzed dissolution in the fractures of carbonate rock when injected with an acidic fluid. One of the criteria examined was the porosity related to loss of mass, which was about 20% higher after contact with the acidic fluid. This loss of mass resulted in a reduction in the shear strength of the sample by 48% based on measurements before and after injection. Each experimental procedure was carried out through the analysis of μCT images to quantitatively estimate the dissolution of the rock fracture.

^18^emphasized the importance of experimental test data for the development of simulation algorithms. Such algorithms are used to improve rock characterization analyses via μCT, which is crucial for enhancing oil extraction. In the study, it is explained that through data obtained by experiments, it was possible to use as input data for simulations of fluid percolation through samples. Microtomographic images were obtained and a 3D reconstruction of the sample images was provided through the μCT technique. The image capture process improved in reliability, allowing porous connections which were previously simulated to be explored with greater confidence.

^19^emphasized the importance of the study of carbonate rocks, explaining their relevance in the oil industry, as well as the structural complexity of carbonate rocks and the challenges surrounding their characterization. Thus, analyses were conducted on samples of coquinas from the Morro do Chaves Formation in the Sergipe-Alagoas watershed, located in northeastern Brazil. These samples were analogous rocks from significant Brazilian reservoirs. X-ray computed tomography was used for the 3D characterization of the rock structures, and the self-organizing maps (SOM) neural network was used to segment the tomographic images. According to the developed tests, μCT proved to be a consistent tool for the qualitative and quantitative analyses of heterogeneous porous spaces, evaluating the porosity, connectivity, and representative elemental volume, and served as an aid to the petrographic characterization of the samples.

In the study by^20^, the μCT technique was used to analyze the porous structure and permeability of a rock artificially cemented with Portland cement. The internal distribution system, size, and connectivity of the pores were characterized.

The objective of this work is the physical characterization of synthetic carbonate rocks and their analysis through the techniques of microtomography and petrography, with a focus on comparative analysis of the internal porous structures of rocks before and after degradation with a reactive fluid. These are essential tools for the characterization of rocks since their analysis produces detailed visualizations of the microstructures of porous media. μCT and Petrography analyses have shown to be fundamental techniques for geological studies of oil and gas reservoir rocks^16^. Also, they can be used for classification of metamorphic degree, and, according to^37^ as a quick and inexpensive tool to predict the mechanical behavior of rocks.

## Methodology

This section presents the experimental protocols performed as part of this research to represent and understand the variations in physical characteristics of synthetic carbonate rocks. The techniques of microtomography and petrography were employed before and after the samples were subjected to an acid flow. The procedure began with the initial characterization of the synthetic rock samples. Following degradation by the fluid, dissolution tests and analyses of the internal structures of these samples were conducted. The characterization of synthetic carbonate rocks consisted of determining the physical properties of the samples, such as porosity and mineralogical distribution, by μCT (analyzed using X-ray Computed Tomography Laboratory [LTC-RX] tomography equipment) and analysis of petrographic slides (analyzed using MEV equipment, electronic microscope of the scan, and a Nikon Eclipse POL) before and after injection of the reactive fluid.

### Synthetic carbonate rock

**Figure 1:**
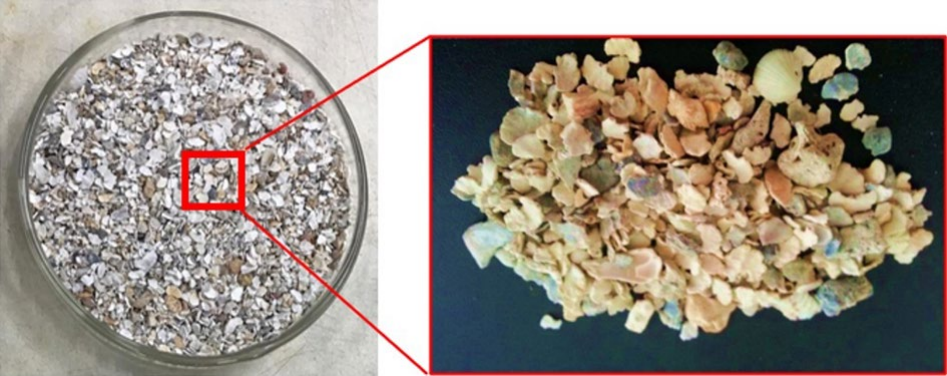
Halimeda grains, shell fragments, and reefs.

Limestone sedimentary formations are predominantly composed of cementations of carbonate fragments, which are made up of ooids, shells, and reefs. The primary material that comprises synthetic rocks is Halimeda (shell remains), as shown in Fig. 1. The average absolute (intrinsic) permeability of the samples is approximately K = 1.02 × 10^–17^ m^2^ (1.03 × 10^–05^ Darcy). Calcite and sand used to produce the rock samples are fine to medium granular materials and have very uniform granulometry, with a uniformity coefficient of 3, according to ABNT NBR 5734/89. The mixture of calcite and sand used to build the rock samples can be considered a granular soil, as 75% have grain diameters of up to 0.149 mm, 17% have grain diameters of up to 0.075 mm and approximately 8% have grain diameters smaller than 0.075 mm.

**Figure 2:**
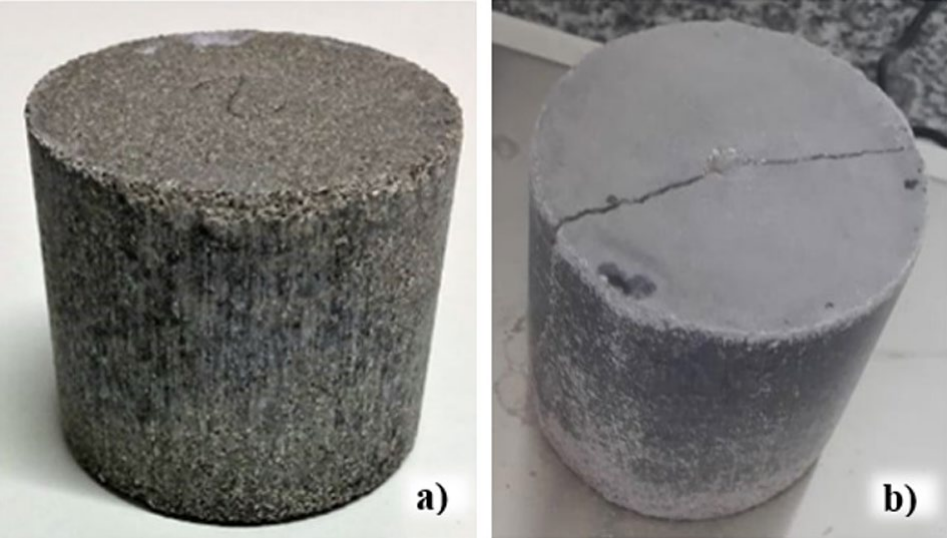
Synthetic carbonate samples. (**a**) Intact rock and (**b**) fractured rock.

Synthetic rocks were produced in the laboratory following the experimental protocol of^21^ and were used in this research to represent and understand the influence of the degradation process on their mechanical, hydraulic, and chemical properties. In addition, the behavior of reservoir rocks subjected to chemical attacks from synthetic samples is frequently studied because control of their characteristics is straightforward. Table 1 shows the percentages of materials that comprised the test samples and Fig. 2 shows the synthetic rocks produced and used in this research.

**Table 1:** Percentages of materials used in synthetic rocks. Reference^21^.

Fraction (%)			
Calcite	Sand	Cement	Water
68	3.6	28	20

To understand the microscopic changes that occurred after contact with fluids, the synthetic samples were developed to achieve a mechanical behavior similar to the behavior of natural soft carbonate rocks. Table 2 describes the values of porosity (*Ø*), void index (*e*), unconfined compressive strength (*UCS)*, tensile compression (*σt*), and modulus of elasticity (*E)* of the synthetic samples.

**Table 2:** Physical and mechanical characteristics of the samples. Reference^21^.

*e*	*Ø* (%)	*UCS σc (*MPa*)*	*σt (*MPa*)*	*E (*GPa*)*
0.58	36.8	11.74	0.33	0.313

### Computerized microtomography

One of the analysis methods used to study the internal characteristics of the materials was µCT. According to^22^, this non-destructive method involves hundreds of microtomographic cross-sections, which produce internal 3D visualizations of the material under study and quantify volume and area data. It is a technique that measures variations in the density and atomic number of materials through the absorption of radiation emitted on the material.

The spatial arrangements of the porosity and mineral distribution directly affected transport processes in the samples. Thus, the μCT technique was a useful tool for the 3D characterization of the spatial mineral distribution of the geological samples. Using this method, high-resolution analyses were performed and, consequently, visualization of detailed microstructures were obtained^23–26^. To characterize the samples using μCT, analysis of their internal matrices was conducted, which provided information related to pore size and distribution within the samples. Information was also extracted about the sphericity of the pores, which was compared to a perimeter sphere, as well as the spatial position of pores in the matrix^27^.

**Figure 3:**
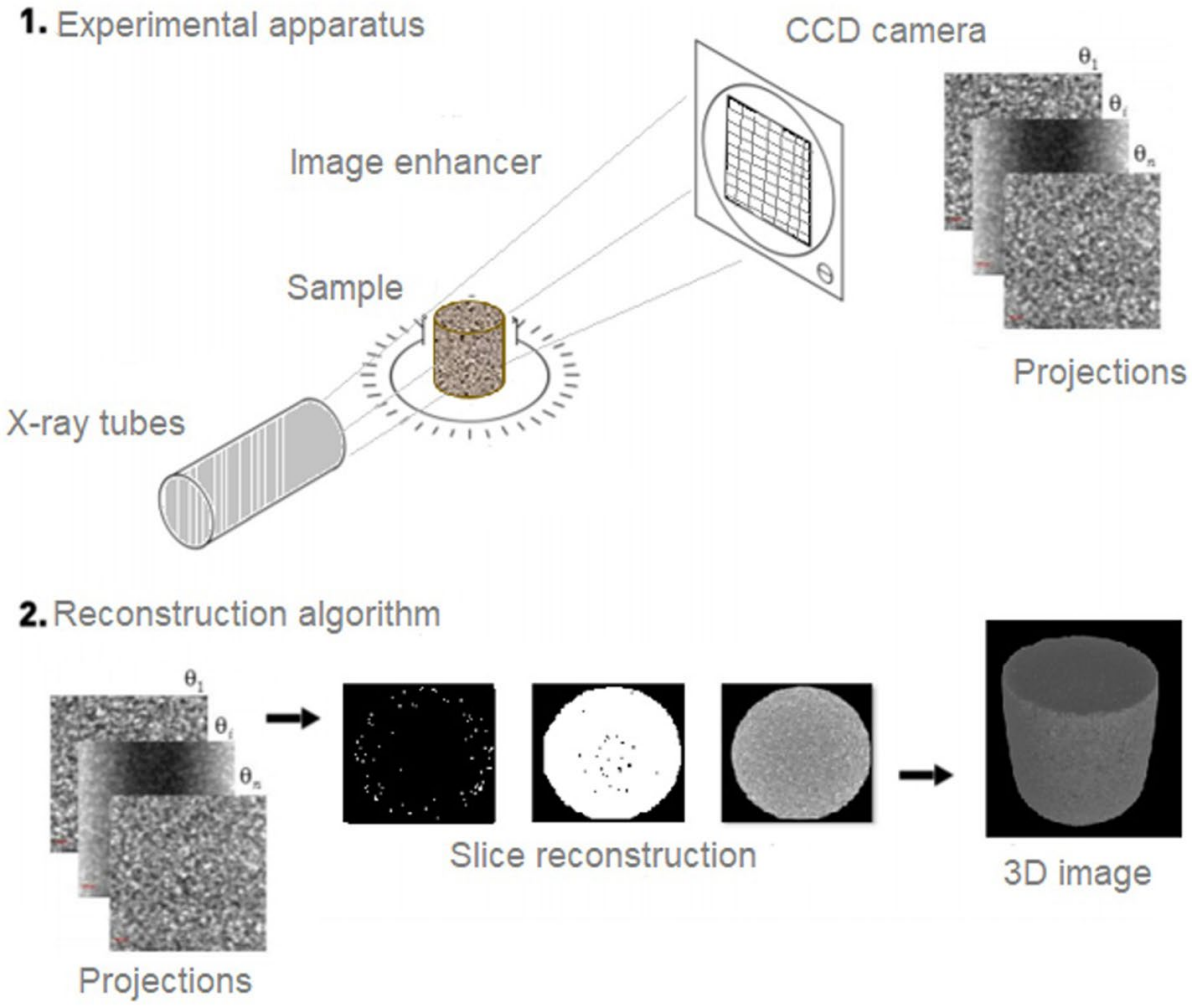
Illustrative diagram summarizing the steps in the acquisition of X-ray microtomography images. Reference: modified^22^.

The material was irradiated from various angles (Fig. 3). Thus, several 2D projections were captured by the charge-coupled device (CCD) camera. Each angle provided a new projection. The reconstruction algorithm was used to analyze the projections and generate a set of slices that were stacked, which provided a 3D image of the verified material^18,28^.

**Figure 4:**
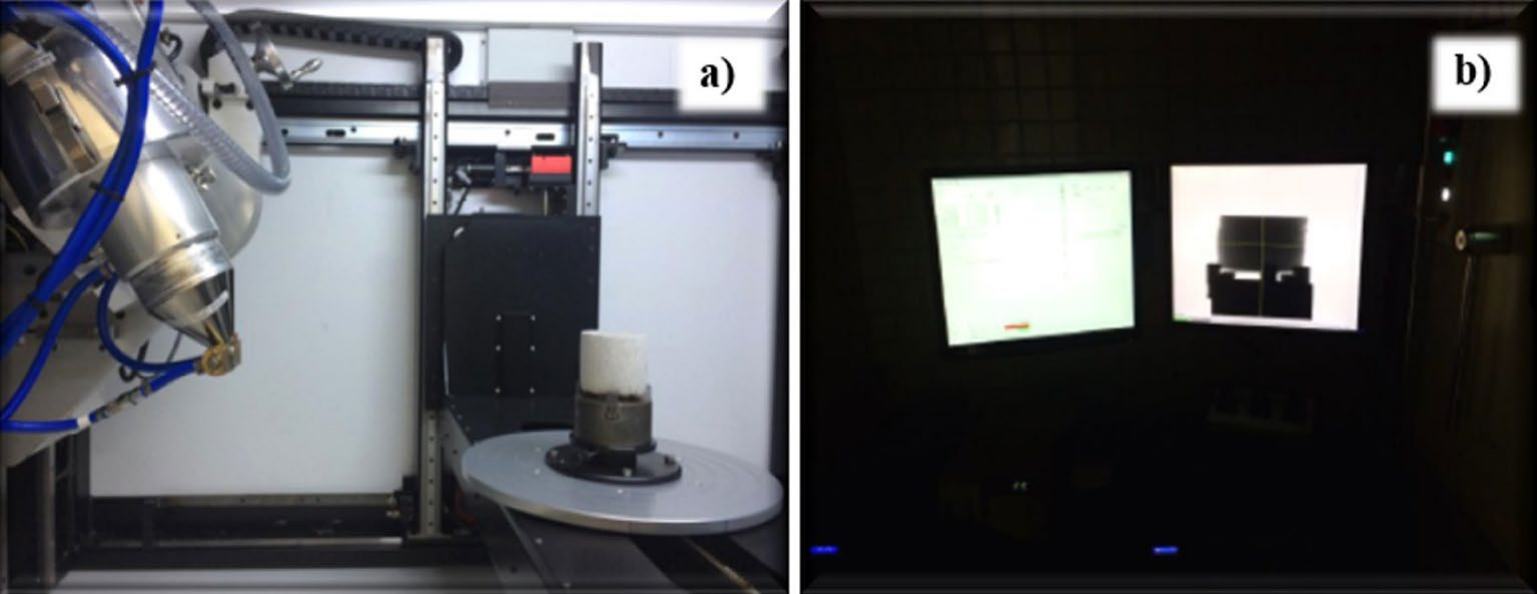
Microtomography equipment from the DEN laboratory. (**a**) View inside the equipment and (**b**) View of the software.

Given the non-destructive nature of the µCT analysis method, this process was conducted before the experimental procedures. The rock samples were forwarded for µCT analysis to compare their porosity data before and after the chemical attack. Scanning was performed at the LTC-RX at the Nuclear Energy Department (DEN) of the Federal University of Pernambuco (UFPE). The microtomography equipment was Nikon’s *X XT H 225 ST*, which used a tungsten tube as the X-ray source with a maximum energy of 225 kV and 1001 µA, as shown in Fig. 4. The samples were mounted such that the X-ray beams were perpendicular to the axis of the cylindrical steel support. A 150 kV and 200 µA energy source with a 0.5 mm copper filter was used to scan the images. According to^29^, this configuration produces images with a satisfactory contrast between the pores, matrix, and rocks.

The programs *CT PRO 3D XT 3.0.3* (for image reconstruction) and *VG Studio* (for treatment of the reconstructed image) were used for pre-processing of the images. The rock samples received around 1200 slices and were exported from *CT PRO* in TIFF format with the appropriate filters (i.e., Gauss filter) for analysis.

Tomography images were also utilized to examine porosity. A methodology to determine the porosity of soils and rocks was developed by a research group of the Soil Image Laboratory at the University of Guelph (Canada) and a group of the LTC-RX of the DEN at the Federal University of Pernambuco (Brazil). For this analysis, the *CTofSoil* plugin from the *image j* program was used. Analysis of the gray level scale in X-ray tomographic images has a partial volume effect called mixed voxels, in which the value is the average of the soil, air, and water interface. Due to limitations, these phases usually overlap each other^29–32^.

To differentiate the interfaces of the images and determine the porosity, the methodology of^29^ was followed. The plugin *Particle Analyze* was used to characterize the pores in detail and perform various levels of analysis. The values used in the *PA* were 0–8 voxels for micropores, 9–1 × 104 voxels for mesopores, and greater than 1 × 104 for macropores^29,33^. The determining the voxels, they were converted to volumes (Eq. 1). To calculate porosity, a ratio between the micro, meso, and macropore data and the total volume of the sample (Vt) was determined, which is the sum of all voxels (converted to volume) in the image (Eq. 2).

1 $${\varvec{V}} = \user2{ N}_{{{\varvec{VOXELS}}}} \left( {\varvec{\delta}} \right)^{3}$$

2 $${\varvec{P}}\left( \user2{\% } \right) = \user2{ }\frac{{{\varvec{V}}_{{\varvec{p}}} }}{{{\varvec{V}}_{{\varvec{t}}} }}\user2{ }100$$

**Figure 5:**
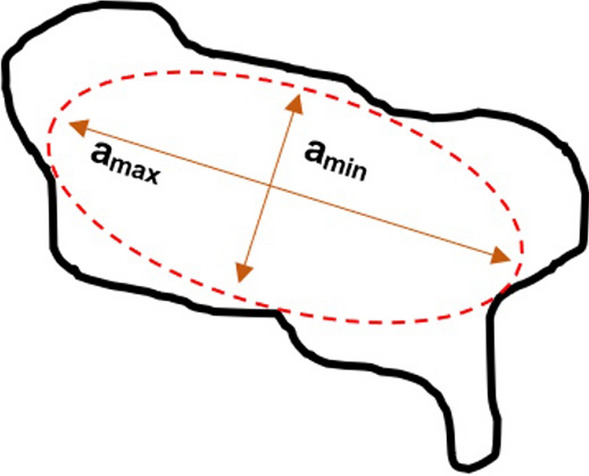
Representation of the ellipse allocation method to obtain orientation properties.

**Figure 6:**
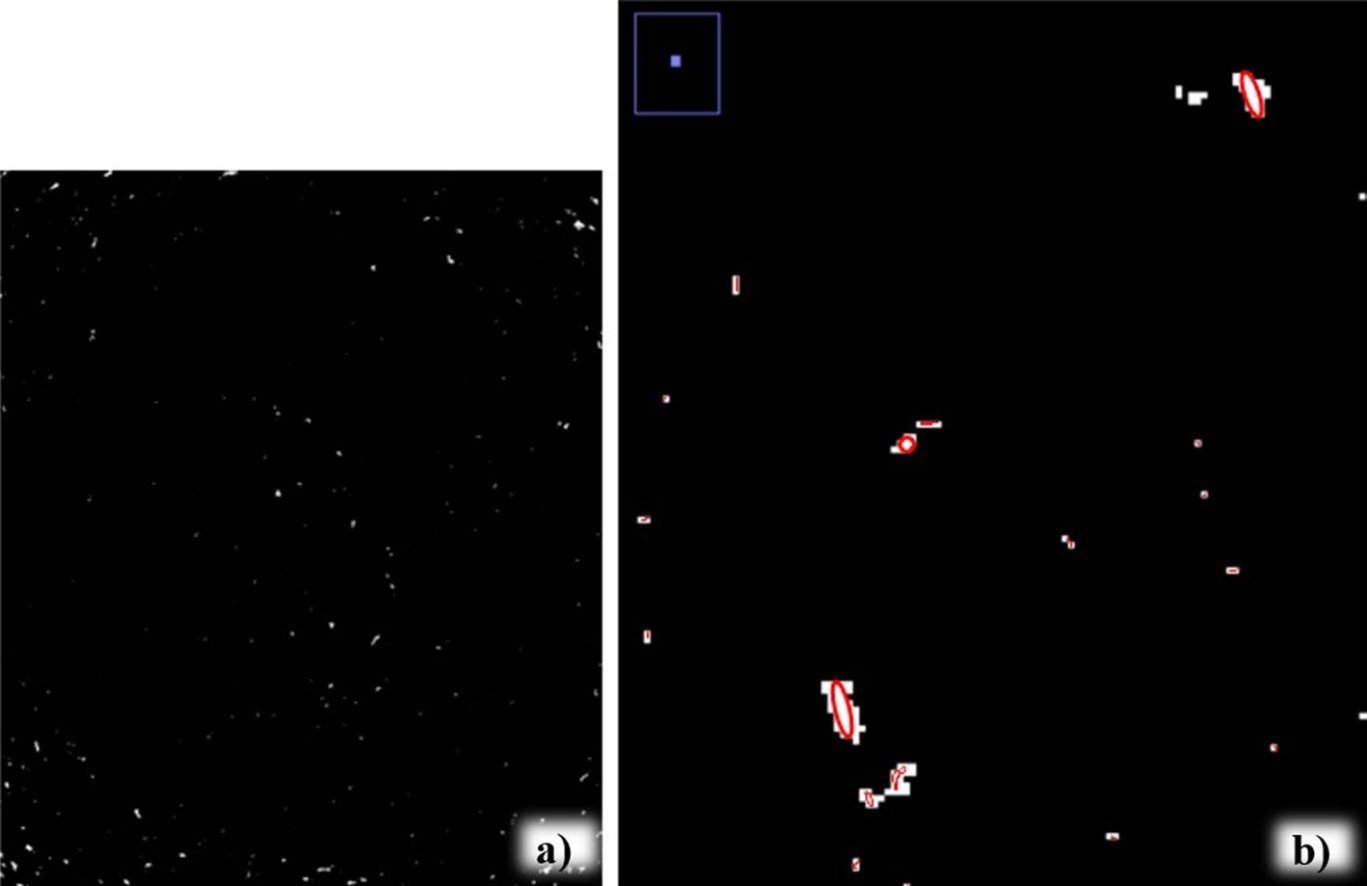
Representation of ellipses for the morphological analysis of each pore. (**a**) Image of the sample with binarization and (**b**) enlarged image to better visualize the ellipses in the pores.

The orientation of the pores is an important property to be explored during rock analysis and was verified using microtomography images. The pore orientation arrangement relates information about the rock structure, such as properties related to stiffness and permeability time anisotropy used in fluid flow analyses. According to^34,35^, one method to determine pore orientation is to apply an ellipse to the image that best represents the region of the detected pore and obtain the orientation of its longest axis (Fig. 5). Accordingly, this procedure was performed for each of the pores identified in the image, as shown in Fig. 6. This simplified the morphological representation of the pores through a geometric approximation. The information generated from the pore ellipse coordinates defined the pores that would be used for the orientation analysis, which quantified the direction of the dominant axis of the pores individually. This analysis can indicate the preferred axis for fluid flow.

### Petrographic microscopy

**Figure 7:**
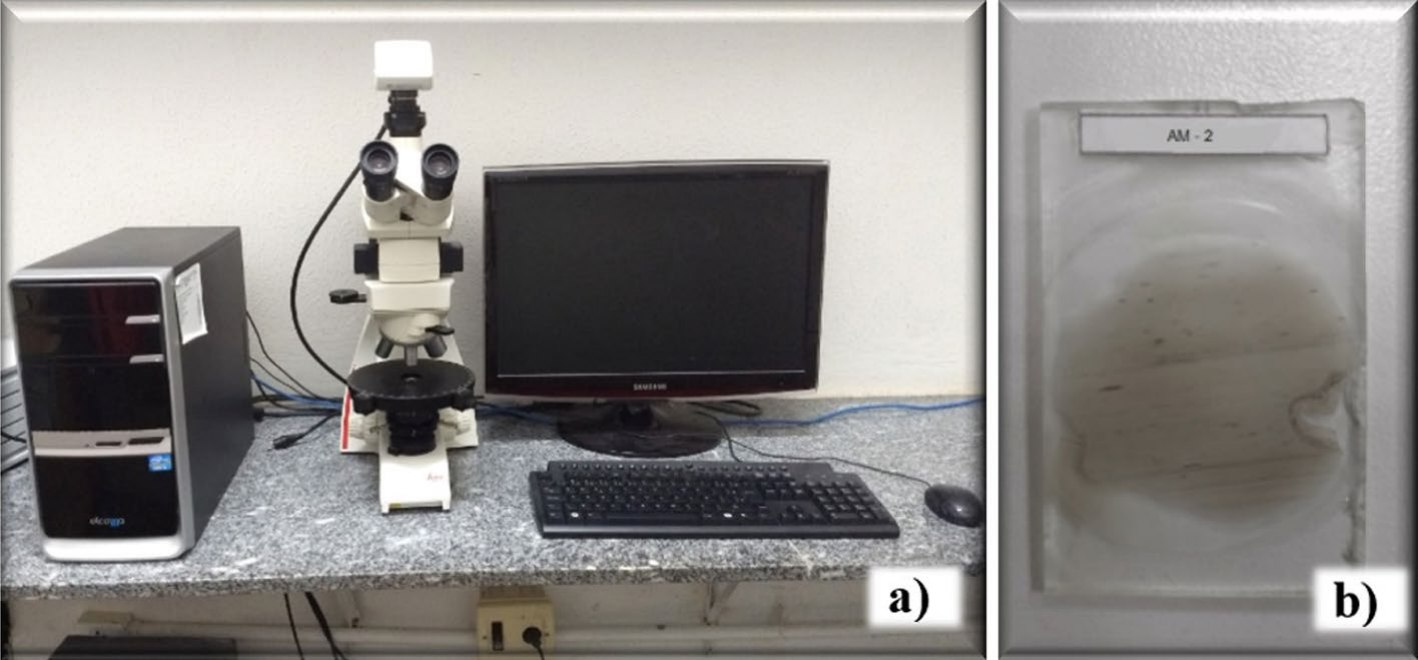
Microscopy procedure using thin slides. (**a**) Thin slides and (**b**) petrographic microscope.

Petrographic microscopy was performed with a petrographic microscope (Nikon Eclipse POL) from the cathodoluminescence laboratory of the geology department at UFPE (Fig. 7a). The procedure for this analysis consisted of extracting a thin sheet with a thickness of 35 µm of the rock to be analyzed (Fig. 7b) and identifying the existing minerals. According to^36^, scanning electron microscopy (SEM) is a tool capable of producing high-resolution images and, depending on the method of image generation, it is possible to visualize the structure with a 3D appearance.

The petrographic microscope is an instrument of fundamental importance in geological studies, whether for studies of reservoir rocks in the search for oil and gas or the classification of metamorphic grades. According to^36,37^, it is an inexpensive tool for quickly predicting the mechanical behavior of rocks. Thus, this procedure was applied to analyze the loss of minerals that occurred as a result of degradation.

### Rock acidification test

**Figure 8:**
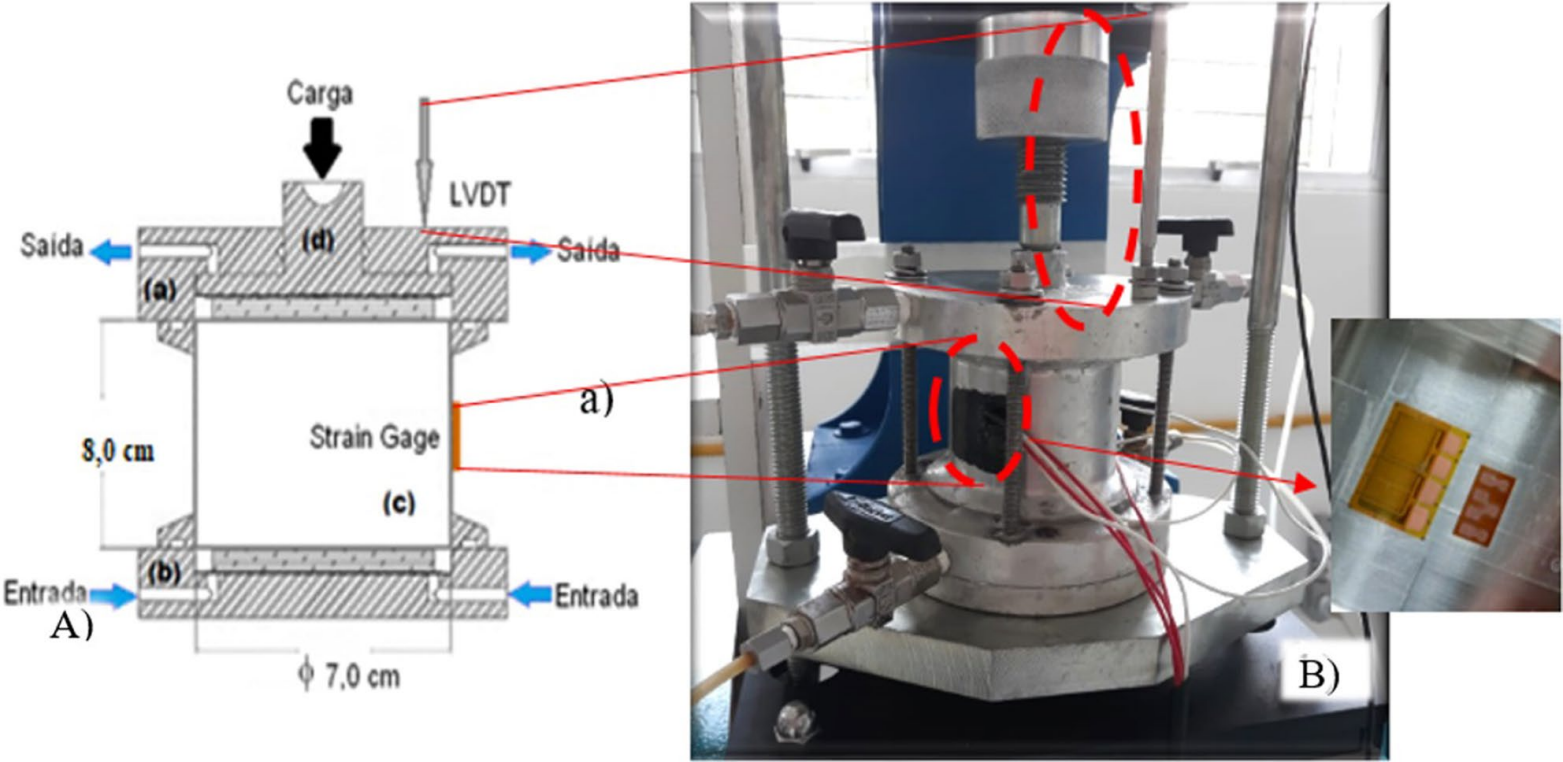
(**A**) Edometric cell diagram comprised of (a) upper, (b) lower, and (c) sample confinement rings and (d) piston for vertical load application. (**B**) Modified edometric cell, highlighting the strain gauge in the confinement ring and the LVDT in the upper part of the cell. Reference: Modified^21,38,39^.

The synthetic samples used for this work were composed of a carbonate material (Halimeda), quartz sand, and Portland Ari cement. To subject the synthetic rock samples to a reactive fluid, oedometer tests were performed using a modified edometric cell with horizontal stress measurements (Fig. 8). High-resolution strain gauges were installed in the middle of the confinement ring of the edometric cell to measure horizontal displacements. For measurements of the vertical displacements, the displacement transducer for linear measurement (LVDT) was used. The samples used for the dissolution test were 6.5 cm in length and 7.0 cm in diameter.

**Figure 9:**
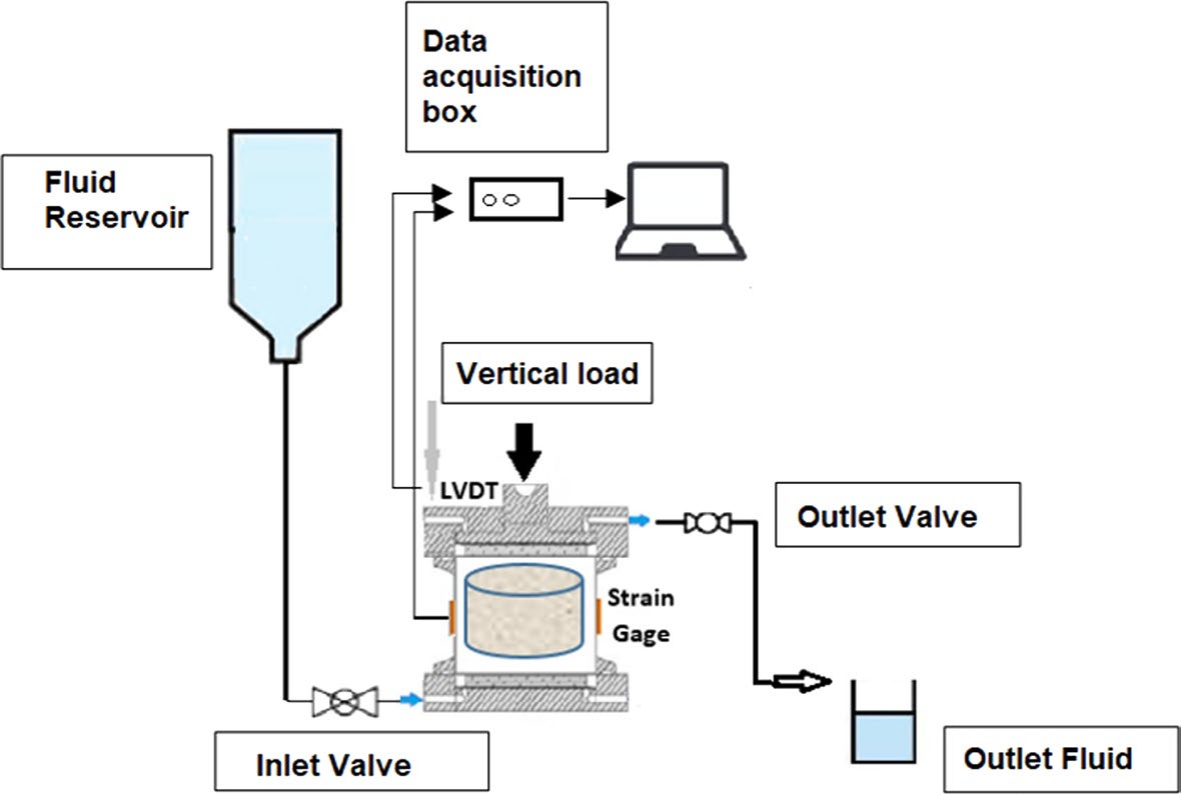
Schematic of the dissolution test experimental apparatus. Reference^21^.

Figure 9 shows the dissolution test scheme. The methodology was composed of the loading and dissolution phases. In the loading phase, vertical loads of 150, 300, and 400 kPa with a duration of 1 h were applied. Then, dissolution began with the fluids of the acetic acid solution at a flow pressure of 12 kPa for approximately 12 h, totaling 15 l of solution injected through the synthetic rock. During the test, pH, permeability, and vertical and horizontal displacements were measured. The acidic solution was collected every 200 ml of fluid percolated in the rock and the permeability was measured. pH was measured with a pH meter during the dissolution.

The dissolution test used an acetic acid solution (chemical formula C_2_H_4_O_2_) with a concentration of 10%, viscosity of 1.22 mPa s, density of 1.049 g/cm^3^ and pH of 2.8. The microscopic characterization analyses of the samples were completed before and after the experimental degradation procedure. Details of the methodology for the dissolution of rocks as well as the output data are described by^21^.

## Results and discussions

In this section, results are presented for the experimental synthetic carbonate rock study to understand the physical changes that occurred after the rock was degraded with an acid flow. A general characterization of the samples’ petrographic structure was conducted as well as a comparison of the structures before and after dissolution. The X-ray microtomography followed the 2D analysis of the samples, resulting in the quantification of the porosity and 3D analysis of the rocks’ structure. In addition to quantifying the porosity, the orientation of the pores in the rock structure was verified and indicated anisotropic permeability.

### Petrographic microscopy

#### Petrographic characterization of thin laminae of synthetic rock samples

Observations from the mesoscopic scale samples (hand samples) are shown in Fig. 11. The samples consisted of an aggregate of minerals cemented by Portland Ari cement, the composition of which has a high percentage of limestone and some oxides. Thus, minerals such as calcium carbonate polymorphs, quartz, feldspar, and minerals from the oxide group were observed in the samples.

**Figure 10:**
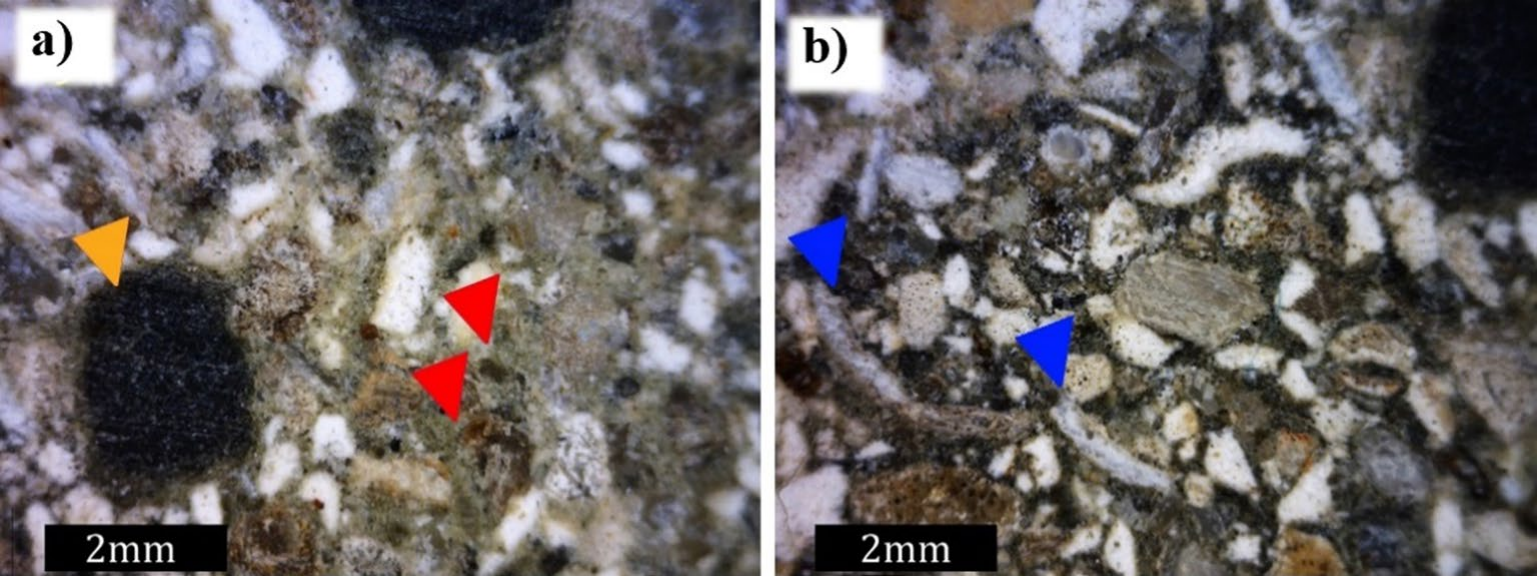
General aspects observed in the Dino-Lite Pro Lupa. (**a**) Faceted quartz grains (red arrow) and round grains related to potential minerals of the oxide group (rutile, ilmenite, and magnetite—orange arrow) and (**b**) carbonatic skeletal grains interpreted as bivalve carapaces (blue arrow).

Calcium carbonate polymorphs occurred in the hand samples as rounded, white grains with coarse sand to silt granulations. In the hand samples, skeletal grains were observed, such as the shells of gastropods, bivalves, sea urchin spikes, and fragments of a green algae group, identified as Halimeda. Quartz occurred as rounded, medium to fine sand grains that were white to transparent in color, and usually exhibited a surface-altering film of iron oxide around the grains. The feldspar grains, on the other hand, have a shape with regular faces, defined by their two preferential breaking planes, or property called cleavage (the tendency of minerals to break up along parallel planes). They were elongated crystals, with a reddish cream color and medium to fine sand granulation (Fig. 10).

**Figure 11:**
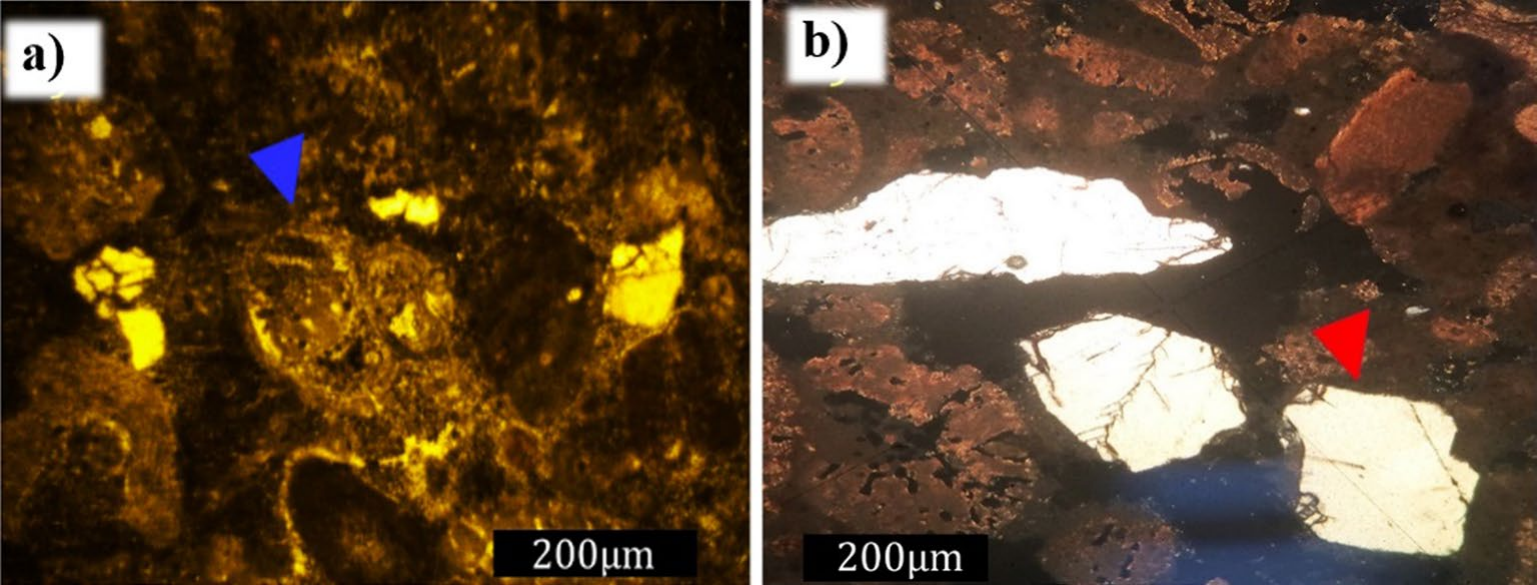
Synthetic carbonate rock observed under the petrographic microscope. Detail of green algae fragment Halimeda (lower blue arrow) and a bivalve carapace (upper blue arrow). Quartz grain (red set) and (b) quartz grains in a pore region (black region, red arrow).

Microscopic features were observed using the petrographic microscope (Nikon Eclipse POL). The framework grains formed predominantly punctual and secondarily concave–convex contact between the grains. There was a predominance of carbonate grains (70%) compared to the siliciclasts (20%) and oxides (10%). At parallel nicols, the carbonate grains were yellowish-white to transparent, and, under crossed nicols, they presented a high interference color characteristic of calcium carbonate, aragonite, and calcite polymorphs^40^. Skeletal grains, mainly fragments of the green algae group Halimeda, indicated by its characteristic internal structure, were also observed under the petrographic microscope, as shown in Fig. 11^40^. Quartz and feldspar grains were transparent to parallel nicols and displayed a gray to orange interference color under crossed nicols (Fig. 7). Oxides were opaque in transmitted light microscopy. The cement used as a binding agent had a brown color to parallel nicols.

**Figure 12:**
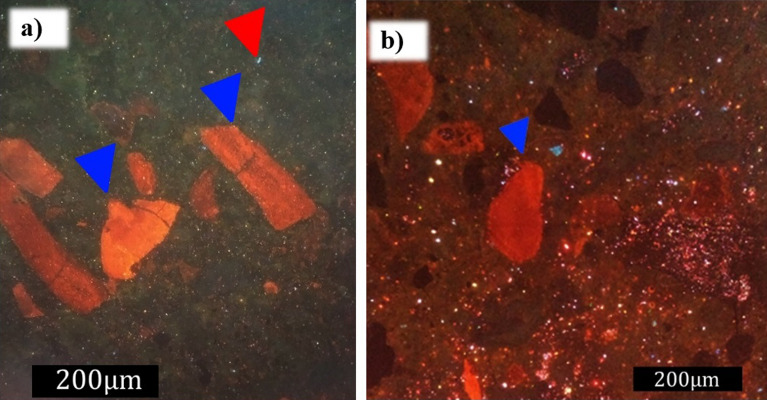
Image obtained from cathodoluminescence. (**a**) Detail for the Halimeda algae fragments showing red luminescence (blue arrow) and fine grains of quartz or feldspar showing a blue luminescence (red arrow). (**b**) Binding cement represented by punctual luminescence located in the matrix of the synthetic rock.

Cathodoluminescence is the luminosity caused by the phenomena of fluorescence and phosphorescence and is emitted by the luminogenic centers of minerals bombarded by an electron beam. Analysis was performed using the same procedure with the microscope model Mk 5; however, using the cathodoluminescence technique^41^ during the petrographic study of the synthetic rock samples, carbonate grains associated with Halimeda algae were luminescence activators. Thus, the carbonate grains demonstrated a characteristic reddish luminescence. Siliciclastic grains, such as quartz and feldspar, exhibited a blue luminescence. The cement used as a binding agent in the synthetic sample showed several luminescence colors, such as blue, yellow, and red, which may indicate the heterogeneous nature of the composition of the cement used (Fig. 12a and b).

#### Petrographic analysis of synthetic rocks before and after degradation

**Figure 13:**
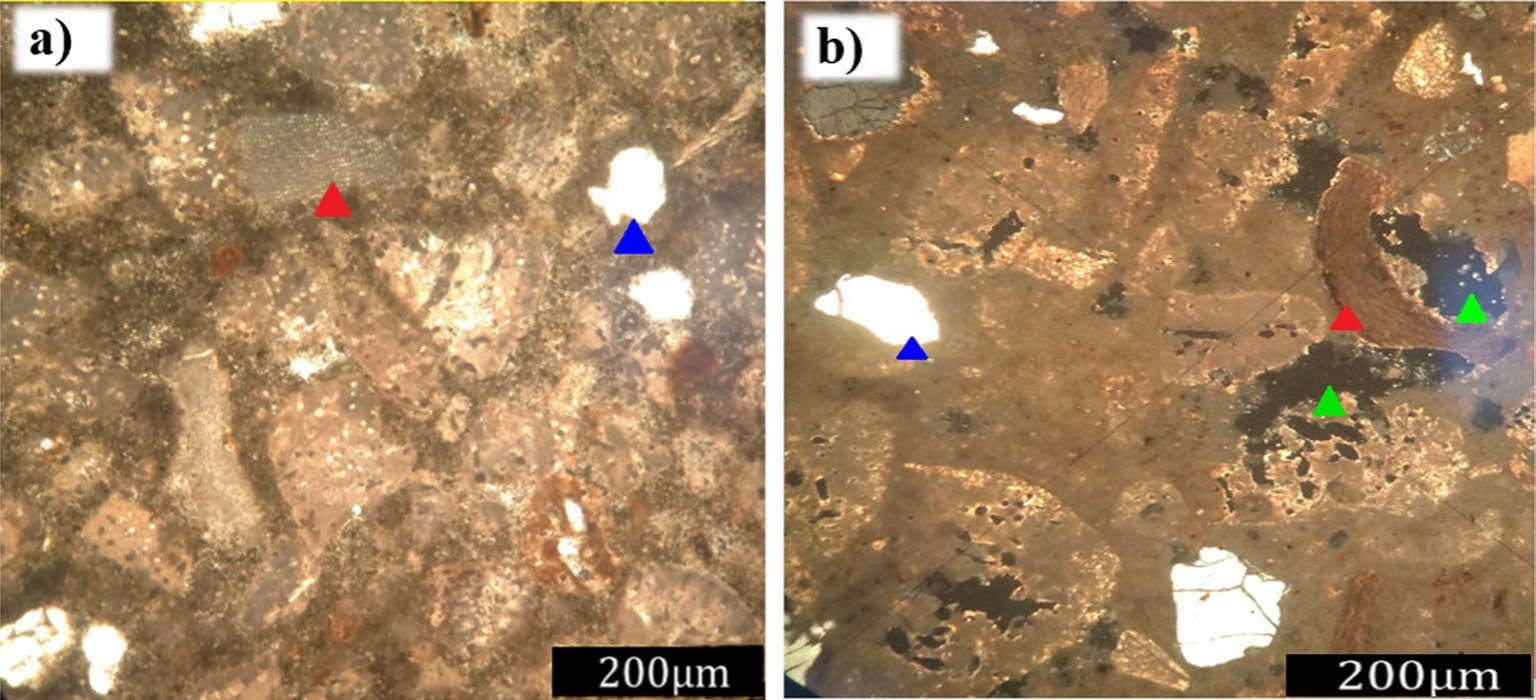
Micrograph obtained using a petrographic microscope (Nikon Eclipse POL) at parallel nicols of the samples (**a**) before and (**b**) after degradation, as well as calcium carbonate skeletal grains of a gastropod (red arrow—carbonate skeletal grain; green arrow—porosity; blue arrow—quartz grain).

**Figure 14:**
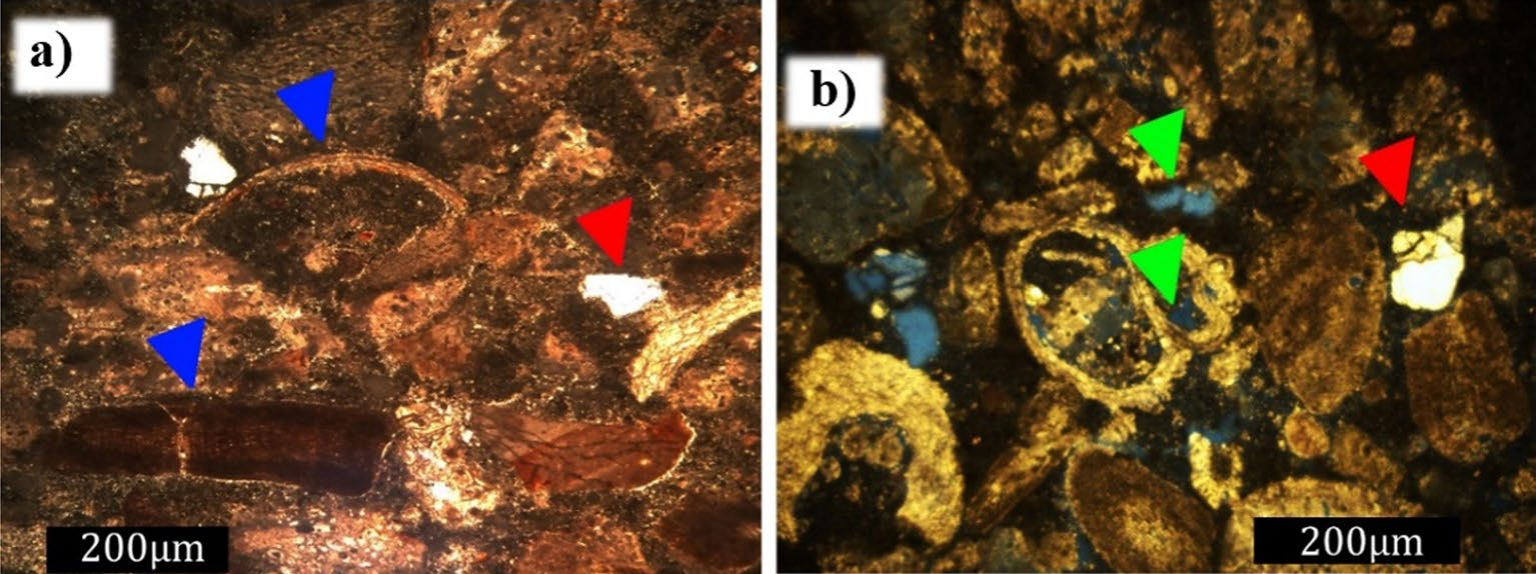
Micrograph obtained using a petrographic microscope (Nikon Eclipse POL) of crossed nicols of the synthetic carbonate rock (**a**) before and (**b**) after degradation, as well as the Halimeda green algae fragment (lower blue arrow) and a bivalve carapace (upper blue arrow).

The synthetic samples had a primary porosity associated with empty spaces both inside and between the grains. Comparing aspects before and after degradation, the primary porosity increased after the passage of the fluid through the synthetic rock. This increase was associated with the inter and intragranular voids, as well as the creation of pores by degradation of the carbonate grains. The difference in the structure of the sample is highlighted in Fig. 13a and b, where the pores are represented by black shading, as well as in Fig. 14a and b, where the pores are represented by blue shading and indicated by the green arrow. The carbonate grains at parallel nicols were yellowish-white to transparent, and under crossed nicols, they presented a high interference color characteristic of calcium carbonate, aragonite, and calcite polymorphs^40^. Skeletal grains were also observed under the petrographic microscope, mainly Halimeda fragments indicated by their characteristic internal structure.

**Figure 15:**
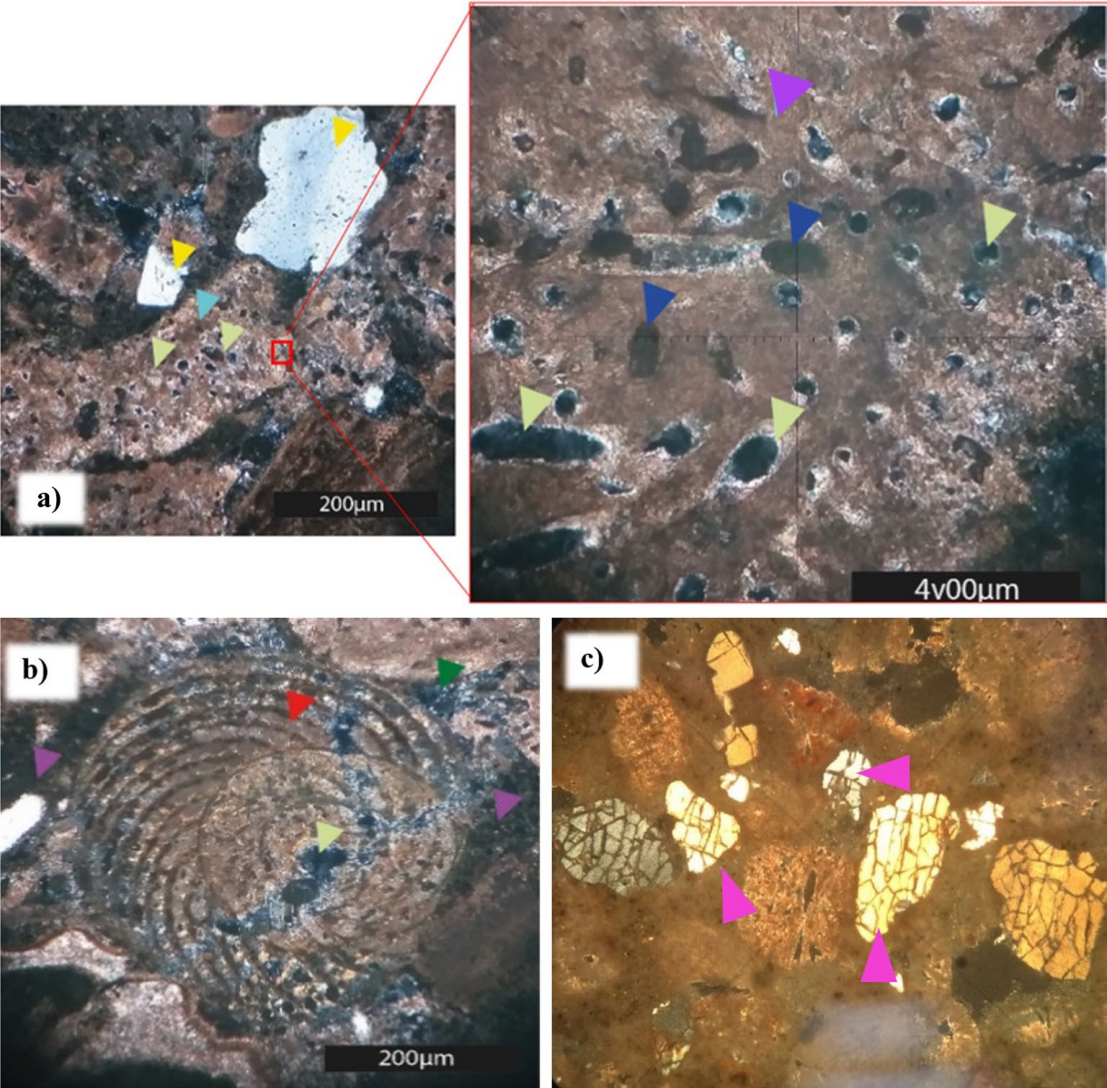
Images (obtained using a petrographic microscope, Nikon Eclipse POL) of the petrographic slide of the cement sample after degradation with longer exposure to acidic fluid, emphasizing the increase in intraparticle porosity. (**a**) Micrograph at parallel nicols (light green arrow—intra-particle porosity; red arrow—carbonate grain; yellow arrow—quartz grain). Enlarged image to more clearly display intra-particle porosity. (**b**) Micrograph of crossed nicols showing calcium carbonate skeletal grain of a gastropod (blue arrow—carbonate skeletal grain; green arrow—porosity; red arrow—rounded grain of quartz; lilac arrow—cement). (**c**) Micrograph at parallel nicols showing changes in the structure of quartz grains after degradation (pink arrow).

An important feature observed during the petrographic analysis was the appearance of intraparticle porosity due to fluid reactivity, as shown in the enlarged image of Fig. 15a. In Fig. 15b, several intraparticle pores by a porosity inside the green algae (Halimeda) are displayed. Another aspect observed was related to cracks in some quartz minerals; as a result of the vertical stress applied to the rock samples, the minerals broke (Fig. 15c). Even though the constituent nature of the quartz minerals was considered to be inert to weathering, the final conditions to which these minerals were subjected caused changes in their structures.

### X-Ray microtomography

#### Characterization of synthetic rocks before and after acidification tests

**Figure 16:**
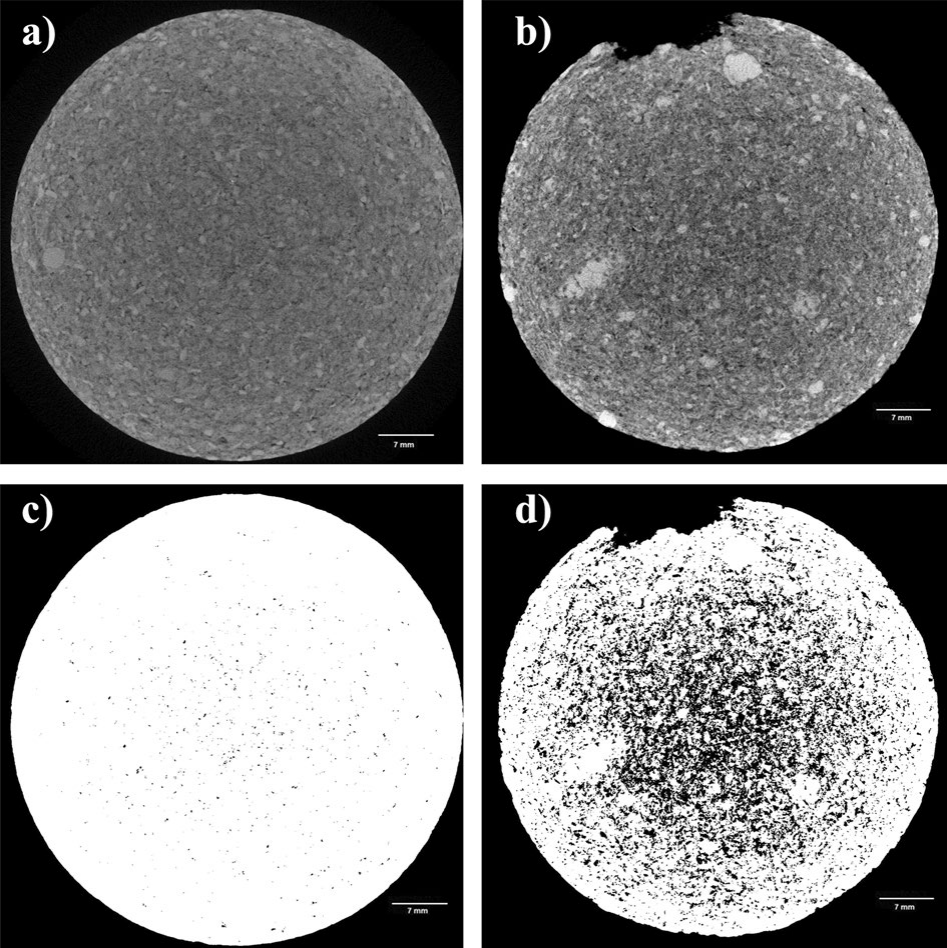
2D analysis of the samples’ tomography images. (**a**) Before rock degradation, (**b**) after rock degradation, (**c**) identification of pores within the rock matrix before degradation, and (**d**) identification of pores within the rock matrix after degradation.

The 2D computed tomography images (Fig. 16) made it possible to observe the increase in porosity that was detected by petrography. An increase in the number of black pixels (representing the pores) between Fig. 16a and b and between Fig. 16c and d can be observed. Thus, there was an increase in the number of pores after the degradation of the samples. By comparing the two samples, it can be verified that the samples degraded with the injection of acidic fluid. The tomography analyses demonstrated that there was a significant increase in the number of pores, indicating that the passage of acidic fluid through the samples caused the dissolution of carbonate minerals that existed in the samples.

All images were treated and any type of artifacts that could affect the analysis were removed.

**Figure 17:**
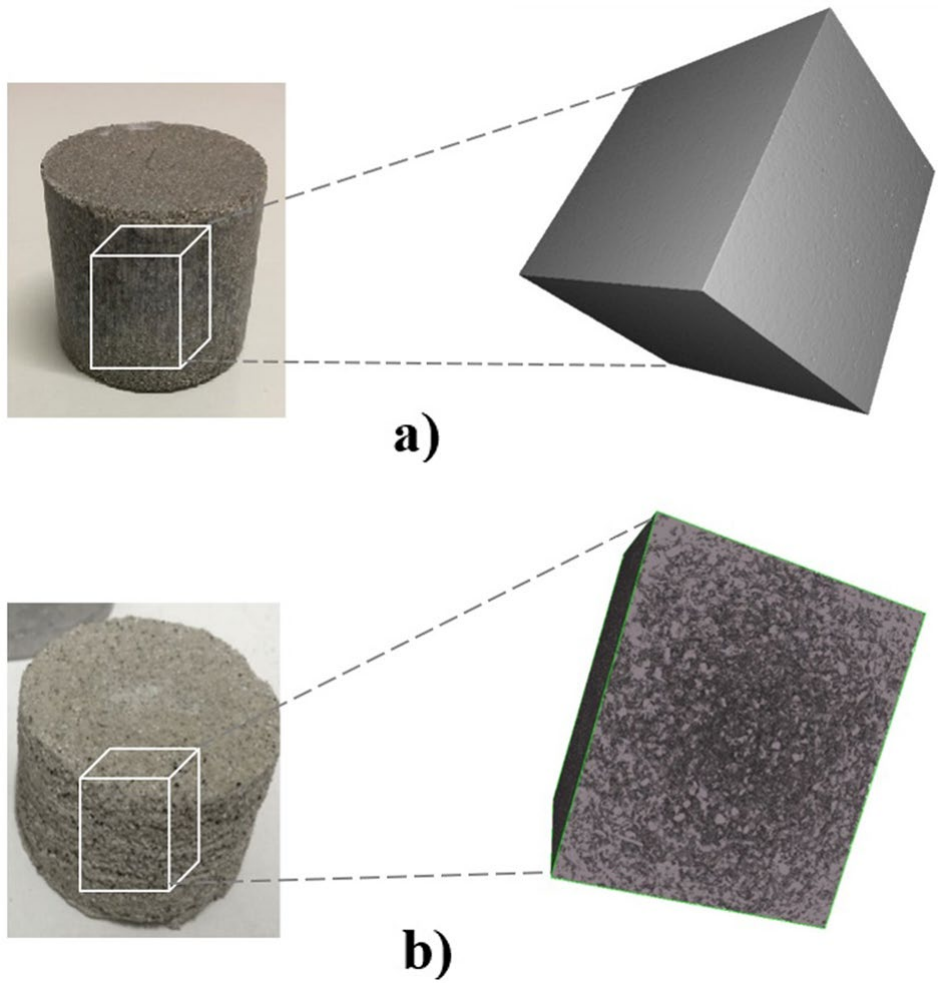
3D structure of the sample's solid matrix with cuts of the sample slices from the central area (**a**) before degradation and (**b**) after degradation.

**Figure 18:**
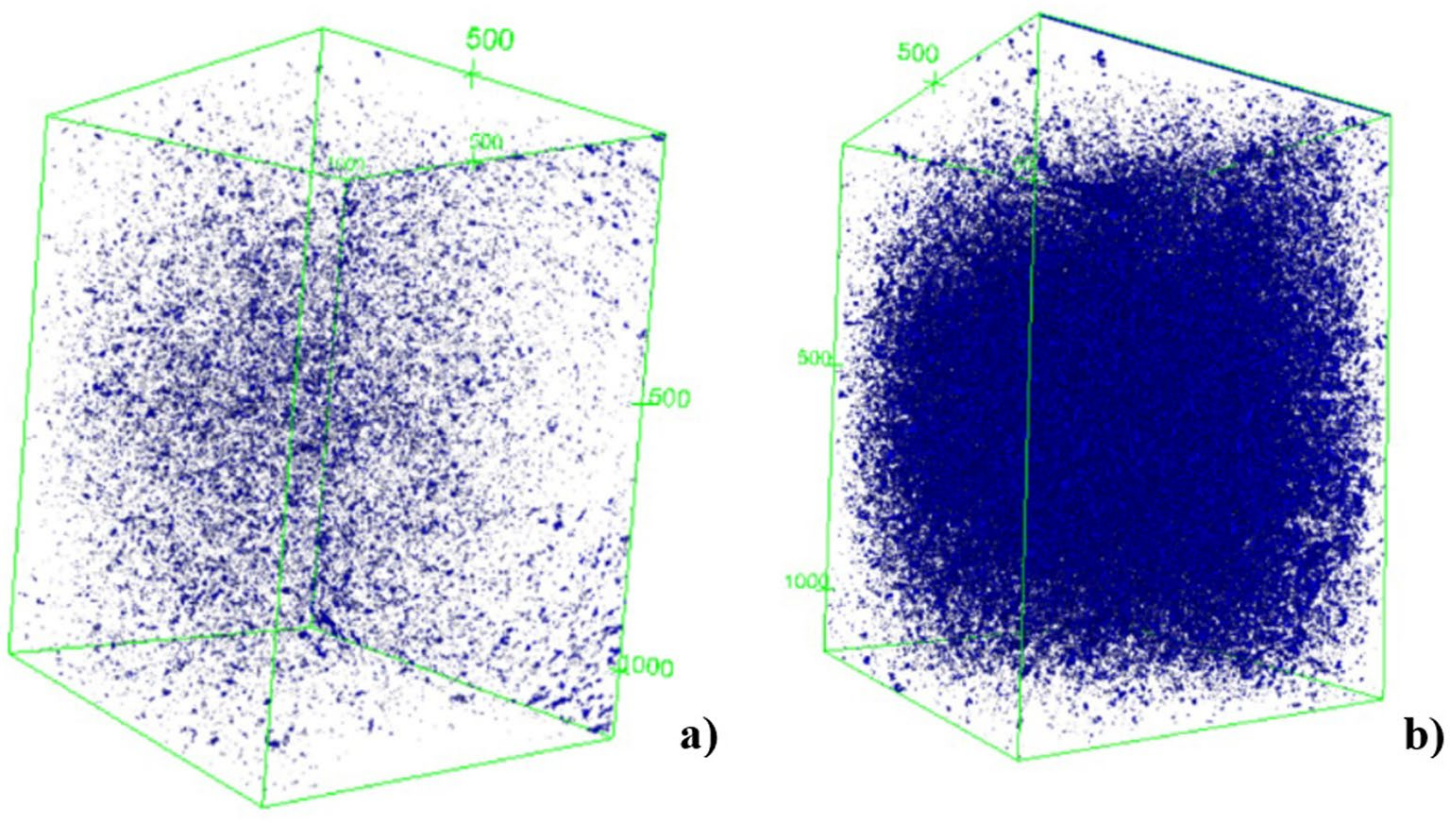
3D structure of the (**a**) pre-degradation rock pore arrangement and (**b**) post-degradation sample pores.

A zone was selected in the middle of the main section of the sample to visualize the spatial structure of the pores as well as to better quantify, analyze, and compare microstructural changes. These changes were attributed to fluid flow, as shown by the representation of the rocks’ study volume in Fig. 17a (rock sample before dissolution) and Fig. 17b (rock sample after dissolution). From the area in the middle of the sample, a 3D construction of the pore disposition and quantification was generated. The structure was uniform and had isolated pores, as shown in Fig. 18a, which represents the distribution of pores in the rock before dissolution. The procedure required binarization of the images; therefore, the accuracy of the results was partially dependent on the user's ability to define the most reasonable threshold level to separate the voids from the solids. After the interaction of the rock with the fluid, the dissolution of soluble minerals occurred and, as a consequence, the pores increased. Figure 18b shows the increase in pore disposition as a result of this degradation.

The porosity values calculated with the standard weighting method (Table 3) are summarized using histogram comparatives along with those determined by μCT 3D imaging, μCT 2D imaging, and laboratory analyses. The 2D and 3D analysis methods are two different types of methodologies, where in 2D the image is analyzed slice by slice and an average of the porosity value is taken and in 3D the sample volume is analyzed by the reconstruction of these slices.That is, despite having similar values in the two analyzes (2D and 3D), the 3D analysis can cover more pores.

**Table 3:** Comparison of porosity values.

Method	Test	*Ø* (%)
3D Micro tomography	Before acidification	31.0
	After acidification	52.0
2D Micro tomography	Before acidification	11.8
	After acidification	41.3
Lab	Before acidification	36.8
	After acidification	53.1

Quantification of the sample porosity was analyzed through the reconstruction of μCT images, which is a non-destructive technique that takes into account the absolute porosity of the samples by quantifying both connected (permeable) and non-connected (closed) voids. Notably, this is an estimated value and, according to^29^, there is a limitation when porosity values are obtained through image analysis when the porosity is lower than the voxel of the analyzed image. Thus, the analysis and quantification of pores via μCT is a more effective tool since it considers the precision of the internal geometric structure of rock samples. However, there is no standard for this methodology, since the materials, resolution of equipment, and filters can vary.

After the pores were counted, morphometric properties were processed based on the area and orientation of the pores. This information was important for understanding the porous structures that comprised the rock samples and made it possible to better understand certain attributes of the rocks, such as compressibility, strength, and permeability^42–51^. Table 4 shows the behavior of the porosity distribution quantification according to the size of the analyzed particles. The image dimensions designate the quality by the number of pixels (x, y) and analyzed slices (z). Taking as a reference the distribution of pores by particle size, the number of large pores was more representative after acidification with the degradation that occurred in the samples. This implies that the grains that were susceptible to degradation were dissolved, which is attributed to the effect on the enlargement of both existing and new pores.

**Table 4:** Behavior of porosity distribution by particle size.

Samples	Pore distribution (%)			
	Image dimensions (x*y*z)	Small pores	Medium pores	Large pores
Before acidification	(948*1128*1311)	0.094	0.723	23.279
After acidification	(948*1128*1070)	0.0067	0.027	48.967

**Figure 19:**
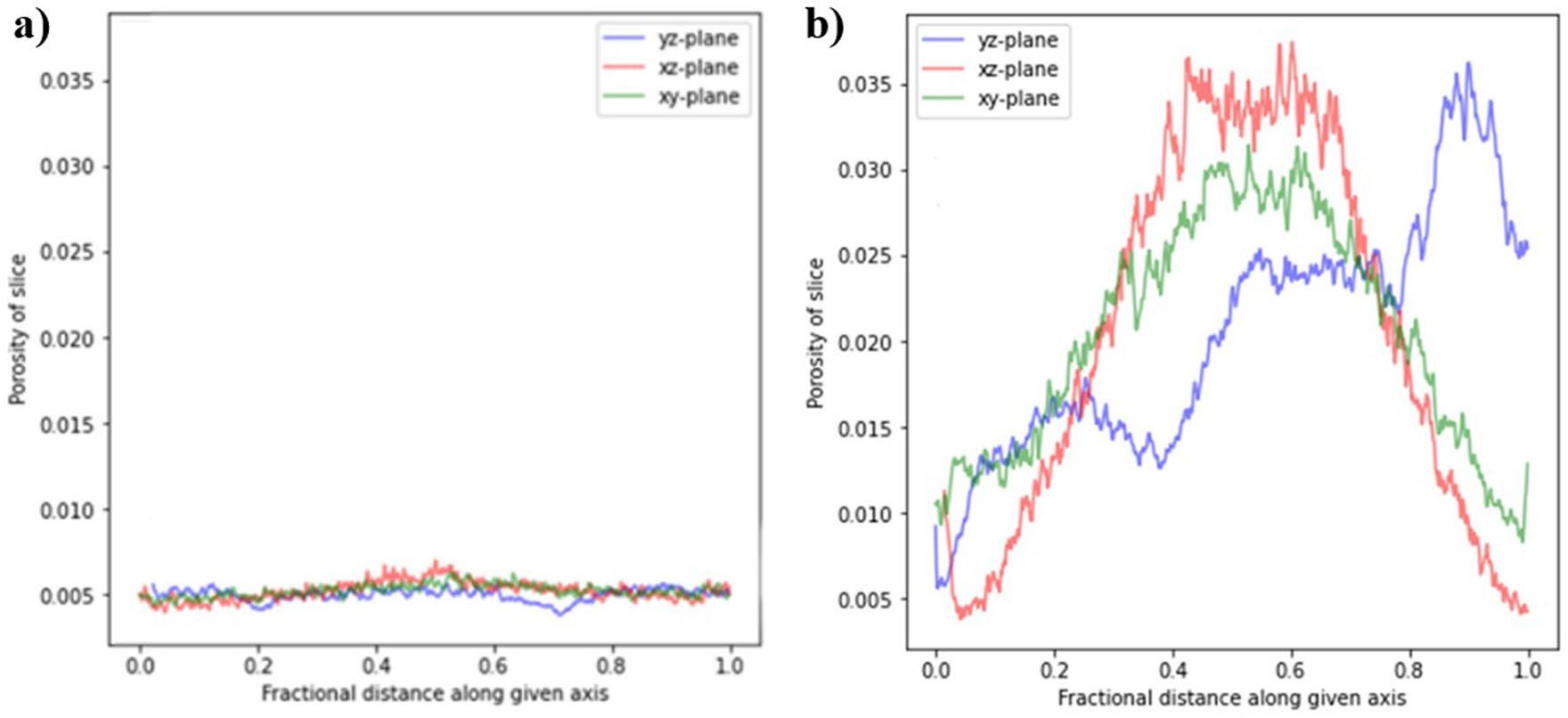
While analyzing pore arrangement before acidification, pore conduct was uniform. (**a**) Before degradation and (**b**) after degradation.

Figure 19 compares the arrangement of the pores and analyzes the preferential path of large pores along the height of the plane of the x, y, and z axes. The xy-plane corresponds to the height of the sample and, in this plane, it is possible to observe that acidification (i.e., the increase in the number of pores) is more expressive in the center of the sample. The xz-plane is one of the lateral planes, which also displays an increase in the number of pores in the center of the sample. In the yz-plane, in addition to the increase in the number of pores in the center, there is an increase on the side, which can be explained by a large pore that dissolved on the side of the sample. While analyzing pore arrangement before acidification, pore conduct was uniform.

**Figure 20:**
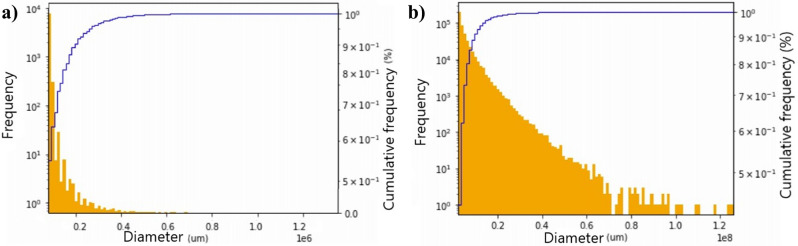
Accumulated pore frequency depending on the area (**a**) before degradation and (**b**) after degradation.

After inspecting the images, data were generated representing the frequency of pores according to their area. It was essential to account for the area of each pore to acquire the pore size distribution and understand the porous structure that comprised the rock sample. Based on the analysis of the pore areas of the rock samples before and after degradation (Fig. 20), the frequency distribution of the pores increased after degradation (i.e., the pore size increased along with the appearance of new pores).

**Figure 21:**
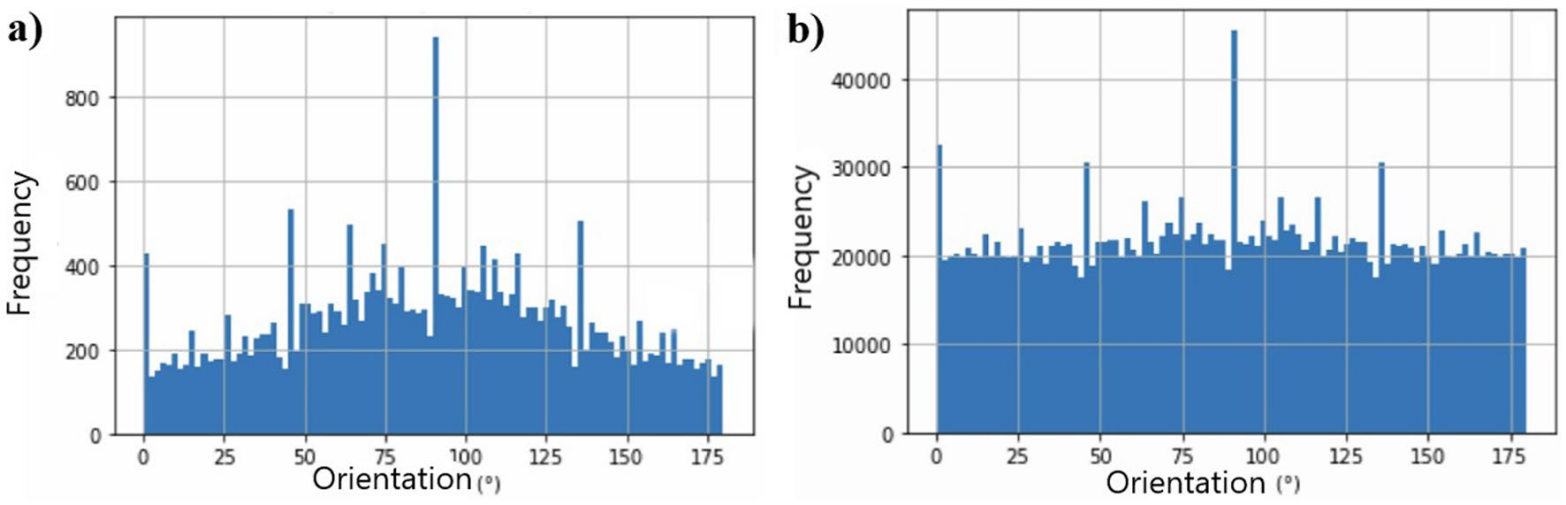
Analysis of pore orientation on the xy-axis (**a**) before degradation and (**b**) after degradation.

**Figure 22:**
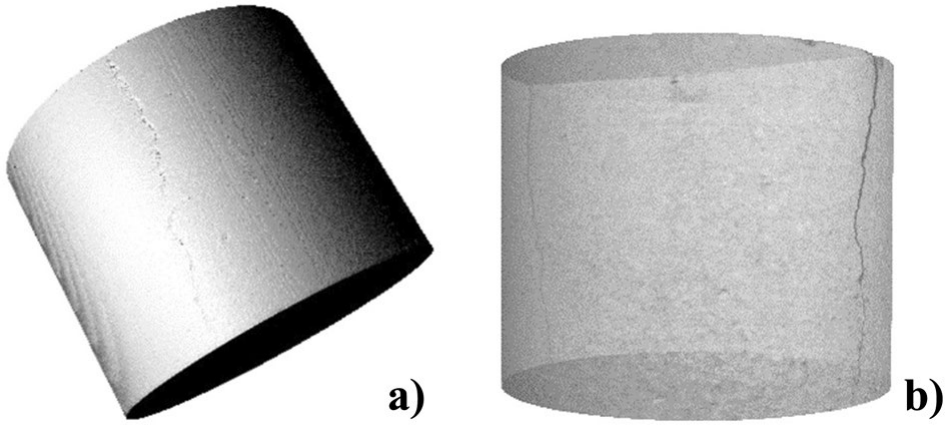
Disposition of the fracture in the sample. (**a**) 3D sample structure and (**b**) 3D sample structure with 50% transparency.

Thus, in the analysis of pore orientation in the xy-direction (i.e., sample height), as shown in Fig. 21, the orientation of the pores was typically higher close to 90°, with maximum frequency values of 800 and 175,000 for samples before and after degradation, respectively. There was also a high frequency at 45° and 175°, which indicated the fluid path. In addition to the appearance of peaks in Fig. 22b, the orientation tended to be uniform.

When the pores exhibited a horizontal (sloping) orientation, a region of greater density was indicated^29,31,51^. The more inclined and resistant the rock, the less permeable. Thus, the analyses allowed the region of the samples that were more porous to be evaluated. The analyses resulted in more accurate estimates of microscopic changes as well as an understanding of how the dissolution of bonds between grains led to macroscopic changes. Such macroscopic changes include loss of strength and mechanics causing irreversible damage to the rock, as observed in the experimental results of^21^.

#### Analysis of induced fractures before and after degradation via μCT

Another analysis conducted using the μCT images was the qualitative study of an induced fracture in the rock. The degradation procedure was the same as that performed on samples without a fracture, and a comparative study of the fracture was conducted before and after the procedure. The purpose of this analysis was to understand the impact of exposure to an acidic fluid on a fractured geomaterial, which was a preferential path for the injected fluid. In Fig. 22, the structure of the studied sample is shown; the fracture was located throughout the diameter and length of the sample. If the sample were torn in half, one face would be 35.20 mm and the other 34.80 mm. The surface profile of the fracture was irregular.

**Figure 23:**
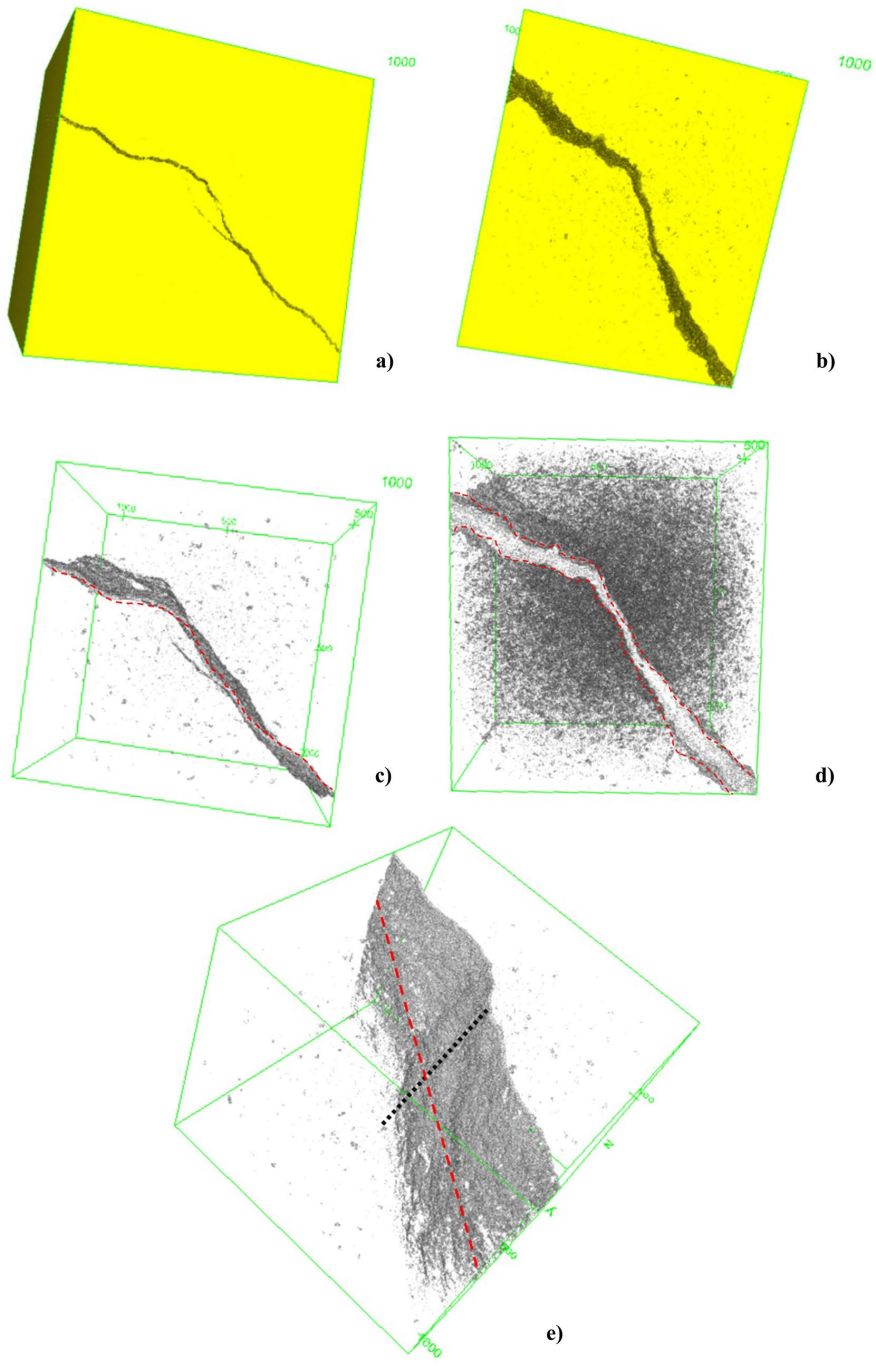
A 3D structure of the solid matrix of the fractured sample (**a**) before degradation and (**b**) after degradation. Comparison of pore arrangement and fracture opening of the specimen (**c**) before and (**d**) after degradation. (e) Fracture length and depth along the specimen represented by the red dashed and black dashed lines, respectively.

Comparing Fig. 23, it is noted that, despite the considerable increase in the amount of pores, the increase in porosity is concentrated in the fracture, since it becomes the preferred flow path. Even with this preferential path, the increase in the number of pores throughout the sample was evident. Preferential paths are also studied in wells, where this effect can be associated with an injection of a reactive fluid. When this happens, there is an increase in porosity in the well, as well as a significant increase in the opening of the well due to the dissolution of soluble minerals. The rock structure close to the well may also be affected.

**Figure 24:**
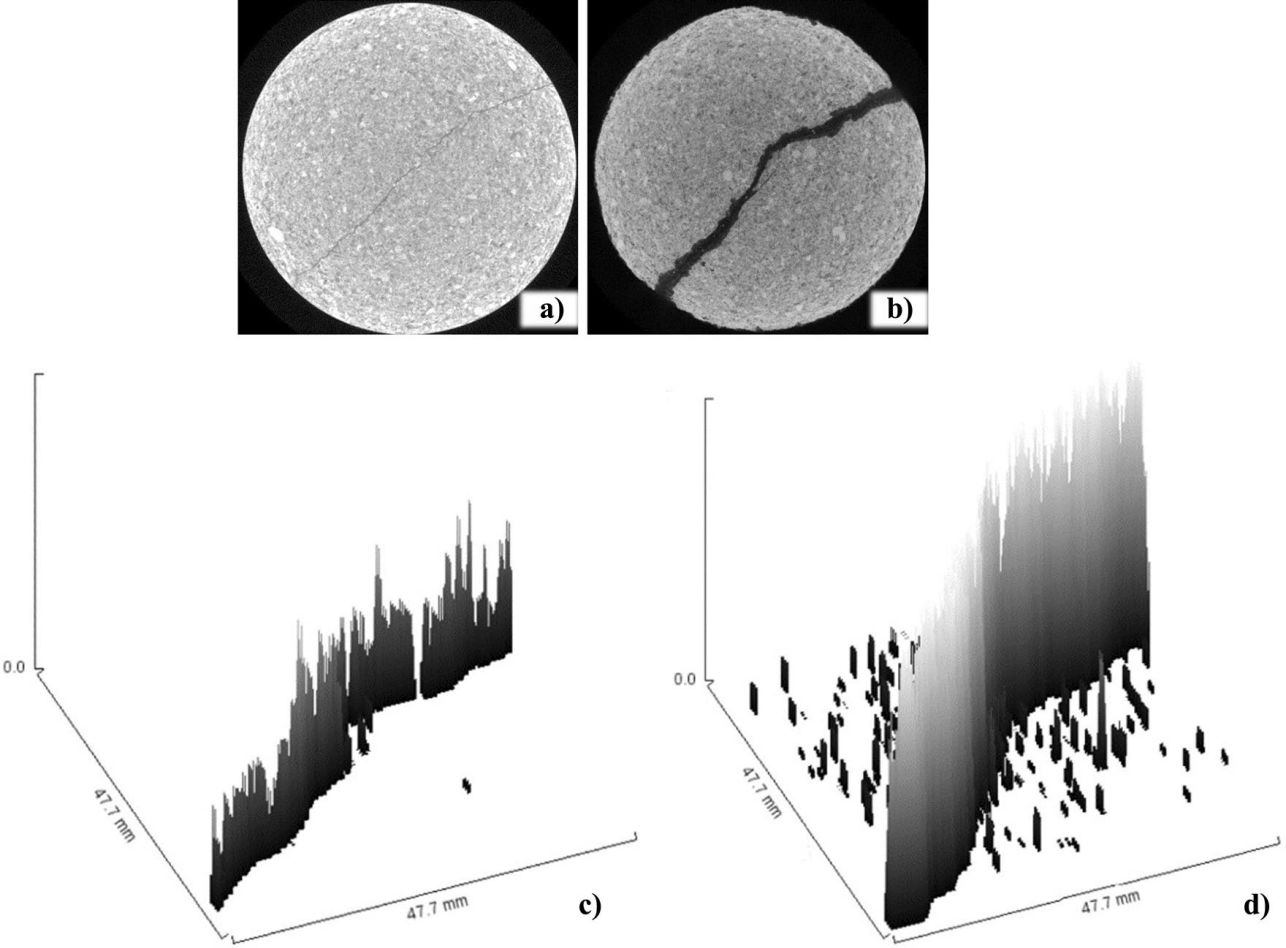
A 2D comparison of sample opening (**a**) before and (**b**) after degradation. Fracture opening distribution along the sample (**c**) before and (**d**) after degradation.

During the reconstruction of the 3D structure of the rocks, the fracture surface was analyzed before and after the passage of the fluid. This analysis is represented in Fig. 24a and b, which show significant increases in the number of pores and in the fracture.

Figure 24c and d present an interpretation of the distribution of significant voids in the samples. Before the passage of fluid, only the fracture at the beginning of the sample was visible. Thus, the fracture was irregular in terms of thickness throughout the sample, having a larger opening at its end compared to the opening at the middle of the sample. Because the opening in the middle of the sample was small, it favored an intact structure. In the analysis after the passage of the fluid, the surface structure of the fracture was visible, both at the end and at the center of the sample.

This procedure was related to the analysis of pore size, in which very small pores (less than 0.1 mm) were omitted from the image. When Fig. 24a is correlated with Fig. 24c, pores with thicknesses from 0.1 mm are visible, and they are even more evident in the fracture region. In Fig. 24d, the pores are more apparent, due to the increase in pore size. However, only those pores with larger dimensions were counted, so there is no way to count the number of pores using this analysis method. To count the increase in the thickness of the fracture, which the dissolution of minerals provided mainly the increase in the fracture opening, by the fracture induce a preferential fluid path.

For a better understanding of the influence of the acid injection process on the fracture, quantification of the fracture opening along the sample was performed before and after the passage of fluid. There was an increase of about 82% in the fracture opening area throughout the sample (Table 5).

**Table 5:** Determination of fracture area before and after degradation.

Sample	Fracture size (pixels)	Fracture size (mm)		
		Top	Middle	Botton
CC—before acidification	0.496	0.56	0.17	0.1
CC—after acidification	2.797	3.96	2.43	2.22

The purpose of this analysis was to highlight the advantages of CT image analysis since it is possible to visualize, determine, and quantify the changes that occur in rocks.

## Conclusions

Non-destructive 3D visualization analysis of the interior of porous material samples is the main contribution that X-ray microtomography provides to petrographic and microstructural studies. Considerable information obtained from petrographic analysis by optical microscopy cannot be reproduced by microtomography analysis. Thus, microtomography is an effective tool in the scope of sample characterization. Analysis of the internal structure of samples via µCT and 3D reconstruction of tomography images has been increasing in popularity. According to^52^, these techniques successfully provided the internal characteristics in similar samples under similar conditions by coupled hydromechanics (i.e., hydromechanics and chemistry).

In addition to 2D analysis, the 3D structure of the pores of the samples before and after injection with fluid was evaluated. The maximum increase in porosity was quantified from 11.8 to 41.3% and 31.6 to 52%, respectively. The values of porosity determined in the laboratory and via µCT image estimation were compared and revealed that rock porosity estimations were more effective using the µCT method. The µCT technique considers the entire sample structure and is a non-destructive procedure. The proposed method quantified the pores according to their area and expressed this information as the pore orientation, providing additional information about the flow’s preferential path. The results of this analysis showed a direct relationship with the obtained reference porosities and, using this methodology, the fracture of the sample was characterized before and after dissolution.

The methodological analyzes of the tomography with these rocks were the starting point, for more detailed analyzes of the porous structure, a tomography with higher resolution should be performed, which is an activity that will be carried out for future publications.

The extraction and injection of fluids at high depths increase mechanical and physical risks due to mineral dissolution as well as changes in pressure, temperature, and saturation that affect the stress state of the reservoir rock. Thus, the analysis of characteristics via microscopic structures is a relevant engineering tool. The conditions to which the rocks are subjected are reflected in the reservoir's acidification processes, such as geological storage of CO_2_ and advanced oil or gas recovery, which pose negative consequences for the material.

This study shows, through analysis of synthetic rocks, what can occur with soft carbonate rocks in contact with acidic solutions. This is important to understand the microscopic behavior of these porous media, serving as a basis for the elaboration of constitutive models to be used later in numerical analyzes of geological media subjected to acidification processes. In carbon capture and storage (CCS) projects, for example, the process of carbonation of water that acidifies the environment during CO_2_ injection requires an integrated analysis of geological modeling (which represents the heterogeneities of the reservoir), as well as the description of the coupled hydro-chemical (and eventually geomechanical (Galindo et al.^21^)) behavior of CO_2_ host formations.

### Recommendations

For future research, it is suggested to produce new carbonatic synthetic rocks, for physical and mechanical characterization.

## Data Availability

All data included in this study are available upon request by contact with the corresponding author.
